# Efficacy and Safety of Acotiamide Versus Mosapride in Patients With Functional Dyspepsia Associated With Meal-Induced Postprandial Distress Syndrome: A Phase III Randomized Clinical Trial

**DOI:** 10.7759/cureus.18109

**Published:** 2021-09-19

**Authors:** Shubhadeep Sinha, Sreenivasa Chary, Pankaj Thakur, Leela Talluri, Mohan Reddy, Gautam S K, Jagan M Mohan, Pankaj Jain, Sunil Naik, Srinivas V C. Reddy

**Affiliations:** 1 Clinical Development and Medical Affairs, Hetero Labs Limited, Hyderabad, IND; 2 Internal Medicine, Shri Ganesh Shankar Vidyarthi Memorial Medical College, Kanpur, IND; 3 Gastroenterology, New Government General Hospital, Siddhartha Medical College, Vijayawada, IND; 4 Internal Medicine, Sterling Hospital, Vadodara, IND; 5 General Medicine, Rajiv Gandhi Institute of Medical Sciences, Srikakulam, IND; 6 Department of Medicine, King George Hospital, Andhra Medical College, Vishakhapatnam, IND

**Keywords:** functional dyspepsia, acotiamide, rome iv criteria, epigastric pain syndrome, postprandial distress syndrome, clinical trial

## Abstract

Background: Acotiamide is a novel prokinetic drug that acts by enhancing the release of acetylcholine and is used in the treatment of functional dyspepsia-postprandial distress syndrome (FD-PDS). Mosapride is indicated to FD-PDS as per the Rome III treatment guidelines. Mosapride 5 mg three times daily (TID) is approved by the Drugs Controller General of India (DCGI) for the treatment of FD-PDS. The objective of this study was to determine the efficacy and safety of Acotiamide in comparison with Mosapride on FD-PDS.

Methods: The 220 patients of either gender (aged 18-64 years) with active PDS included in the study were centrally randomized 1:1 to receive either 100 mg Acotiamide (test product) or 5 mg Mosapride (reference product) TID for four weeks. Responder rates for the overall treatment effect (OTE) at the end of four weeks were the primary efficacy endpoint. Secondary efficacy endpoints included the elimination rate of postprandial fullness, upper abdominal bloating, and early satiation. The study also evaluated the OTE at each week, individual symptom scores, and quality of life (QoL) assessed by the Short Form-Nepean Dyspepsia Index questionnaire (SF-NDI). The safety endpoints included assessments of treatment-emergent adverse events (TEAEs).

Results: At the end of four weeks, the responders in the Acotiamide versus Mosapride group for OTE was 98% versus 93.27% in the per-protocol (PP) population. Among the intent to treat (ITT) population, the comparison of Acotiamide versus Mosapride stood at 95.15% versus 89.81%. Secondary efficacy endpoints were significantly improved with 100 mg TID Acotiamide, which was evident from the improvement in postprandial fullness (14.56%), upper abdominal bloating (15.53%), early satiation (10.68%), and QoL (13.7 ± 4.67).

Conclusions: Our study results demonstrated that Acotiamide is effective, safe, and well-tolerated and had significantly improved the QoL over a four-week treatment period in FD-PDS patients. The efficacy and safety profiles of Acotiamide were similar to Mosapride.

## Introduction

According to the ROME III and the updated ROME IV criteria, functional dyspepsia (FD) is defined as the presence of one or more symptoms related to the gastroduodenal region. FD symptoms include postprandial fullness (PPF), early satiety (ES), epigastric pain, and epigastric burning in the absence of any underlying organic, systemic, or metabolic disease [1,2]. Globally, the prevalence of FD varies between 5% and 11% [3,4].

The pathophysiology of FD remains to be completely understood. Causes of FD could be multifactorial and may include dysfunction of sensory and motor neurons of the gastrointestinal tract, immune-related dysfunction, dysbiosis of the gut microbiome, and gut-brain axis dysfunction [5]. In addition, predisposing factors for FD also include infections with bacteria like *Helicobacter pylori*, *Escherichia coli O157*, *Campylobacter jejuni*, and* Salmonella*. Other risk factors for FD are long-term antibiotic use, therapy with non-steroidal anti-inflammatory drugs, obesity/overweight, smoking, and people with psychosocial disorders. Patients with FD are therefore treated with different classes of drugs including the proton pump inhibitors (PPIs), H_2_ receptor antagonists (H2RA), prokinetic agents, and antidepressants [6]. A recent study from Japan had recommended the use of Rikkunshito, herbal medicine, to treat FD as an alternative to the prokinetic drugs currently in use [7].

Mosapride is a 5-hydroxytryptamine (5 HT4)/serotonin receptor agonist that is widely used in the treatment of FD [8]. Mosapride facilitates both gastric and colon motility and increases gastric emptying by promoting the release of acetylcholine at nerve terminals in the mesenteric plexus [9,10]. Acotiamide, a prokinetic drug, has received its first approval in Japan for the treatment of epigastric bloating and ES in FD patients. Acotiamide exerts its activity by inhibiting the acetylcholinesterase (AChE) enzyme and resulting in enhanced release of acetylcholine at neuromuscular junctions [4,11]. This causes an increase in the force of muscle contractions and enables gastric emptying. Acotiamide acts directly on the gut and through the brain-gut axis involving the central nervous system [12]. A similar prokinetic drug, cisapride, was withdrawn from the market, and also there are limitations for the usage of other drugs with prokinetic action such as domperidone and metoclopramide. There is no drug of choice for safe and efficacious long-term usage against meal-related symptoms of FD [13]. Mosapride is an efficacious, safe, and widely used drug, and its prokinetic effect is similar to Acotiamide. Furthermore, the choice of the comparator drug, Mosapride, in this study was also made based on the recommendations of the subject expert committee (SEC), Central Drugs Standard Control Organization (CDSCO), India.

The available literature suggests several limitations concerning the therapeutic approaches to treat and manage patients with FD. Among them, limited data on the long-term efficacy and safety of drugs used to treat FD assumes increased significance. Also, it is important to understand the potential adverse effects of various therapeutic agents.

Although both Mosapride and Acotiamide are used by clinicians to treat FD patients in India and elsewhere in the world, currently, there is no consensus with regard to the performance, efficacy, and safety of both drugs in relieving the symptoms associated with FD. Therefore, this phase III clinical study was carried out to evaluate the efficacy and safety of Acotiamide in comparison with Mosapride, in Indian adult patients with FD.

## Materials and methods

Study design

This multicenter, randomized, active-controlled, parallel-group, assessor blind, phase 3 study was designed to evaluate the efficacy and safety of Acotiamide in comparison with Mosapride in patients with FD-PDS. The choice of the comparator drug was made based on the recommendations of the SEC, Gastroenterology. Also, the CDSCO, India, suggested the use of comparator drugs belonging to the same drug class and having similar prokinetic effects. The study was conducted at nine investigative sites in India from March 2016 to August 2016. The trial was registered with Clinical Trial Registry-India (CTRI/2016/03/006763) before enrolment of the first patient in the study. The study was initiated after obtaining approval of CDSCO and IRB/IEC of each participating study center.

Inclusion and exclusion criteria

Patients of either gender aged ≥18 years to ≤64 years and diagnosed with FD-PDS as per the Rome III classification were included in the study. Patients with only epigastric pain syndrome were included if the symptoms causing distress were meal-related at the time of enrolment. All the patients underwent upper abdominal endoscopy at the time of screening to rule out any abnormalities in the esophagus, duodenum, or stomach.

Patients with a history of heartburn within 12 weeks; diabetes mellitus requiring treatment; severe abnormality in the electrocardiogram; serious depression or anxiety disorder; biliary tract disease and/or pancreatitis; irritable bowel syndrome; presence of any symptom indicating serious or malignant disease; clinically significant metabolic, hepatic, renal, or hematological disorders; and drug or alcohol abuse were excluded. After the baseline period, anti-secretory drugs, prokinetics, antacids, non-steroidal anti-inflammatory drugs, and antidepressant drugs were not allowed.

Randomization

The study comprised a maximum of one week of screening and baseline period followed by four weeks of the treatment period. During the baseline period, patients were given diaries to mark their symptoms with severity on each day to determine the study eligibility. Randomization was performed based on a computer-generated randomization sequence and by using statistical software. Eligible patients were assigned a unique patient ID and treatment regimen given in the ratio of 1:1. The patients in each group received either Acotiamide 100 mg (test product) or Mosapride 5 mg (comparator drug) three times daily (TID) as per the central randomization schedule followed throughout the study centers.

Efficacy and safety endpoints

Responder rates based on overall treatment effect (OTE) by using a seven-point Likert scale at the end of treatment visit was the primary efficacy endpoint [14]. On the OTE scale, patients with “extremely improved” or “improved” were considered as responders. Secondary endpoints included OTE by using a seven-point Likert scale at each week; elimination rate of upper abdominal bloating, postprandial fullness, and ES at the end of treatment visit; the improvement of individual symptoms score on a severity scale of 0-3 (none, mild, moderate, and severe) at each week and by using Short Form-Nepean Dyspepsia Index questionnaire (SF-NDI) for improvement in disease-specific quality of life (QoL) [15]. Safety variables included all treatment-emergent clinical and laboratory adverse events (TEAEs).

Statistical analysis

The sample size was calculated considering a responder rate (OTE) of 52% of the reference product (as per published literature) and assuming no difference in the proportion of OTE between test (Acotiamide 100 mg) and reference (Mosapride 5 mg) products. To confirm the non-inferiority of test (Acotiamide 100 mg) product to reference (Mosapride 5 mg) product, with 80% power, 0.025 level of significance, and a non-inferiority margin of -20% of the control value, a sample size of at least 99 evaluable patients were required per group, i.e., about 198 patients. Assuming a dropout rate of approximately 10%, about 220 subjects were required to be enrolled in the study with a 1:1 treatment allocation ratio in test and reference arms. All continuous demographic parameters were summarized using numbers, mean, median, standard deviation, range, and quartiles. Fisher’s exact test was used to compare proportions like males/females. The primary and secondary endpoints were analyzed using the Chi-square test and comparison between groups by using t-test. Adverse events were coded using Medical Dictionary for Regulatory Activities (Med DRA Version 19.1). The incidence of serious adverse events was compared across the treatment groups using Fisher’s exact test. SAS® Version 9.4 (SAS Institute Inc., USA) was used to perform all statistical analyses.

## Results

Patient disposition and characteristics

**Figure 1:**
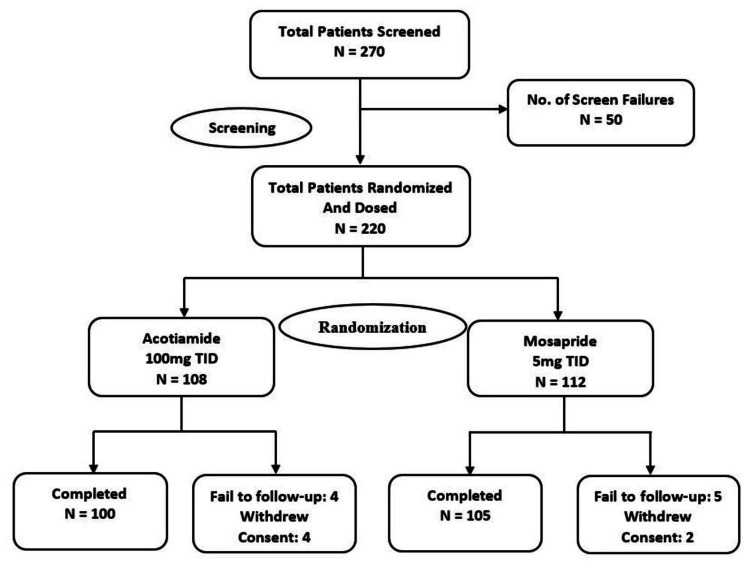
Patient disposition

A total of 220 patients with FD-PDS as defined by the Rome III criteria were enrolled and randomized in a 1:1 ratio to receive either Acotiamide 100 mg (n = 108) or Mosapride 5 mg (n = 112) TID before meals (Figure 1).

ITT population consisted of 218 (99.1%) patients (107 [99.1%] in the Acotiamide group and 111 [99.1%] patients in the Mosapride group, respectively), and PP population consisted of 204 (92.7%) patients (100 [92.6%] patients in the Acotiamide group and 104 [92.9%] patients in the Mosapride group, respectively). Safety analyses were conducted for all enrolled patients. The demographic characteristics of patients were similar between both treatment groups and are summarized in Table 1.

**Table 1:** Demographic and baseline characteristics of the patients Data are shown as mean ± SD or n (%). *p values are obtained by performing Fisher’s exact test. **p values are obtained by performing a t-test.

Characteristics	Acotiamide (N = 108)	Mosapride (N = 112)	p-value
Gender			
Male	2 (1.9%)	3 (2.7%)	1.0000*
Female	106 (98.1%)	109 (97.3%)	
Age (years)	38.1 ± 11.44	37.8 ± 11.56	0.8567**
Height (cm)	161.40 ± 8.02	162.01 ± 8.10	0.5749**
Weight (kg)	59.61 ± 9.31	60.26 ± 10.65	0.6306**
BMI (kg/m^2^)	22.865 ± 3.227	23.006 ± 4.040	0.7765**

Primary efficacy endpoint

The responder rate was 98% and 93.27% (PP population) and 95.15% and 89.91% (ITT population) in the Acotiamide and Mosapride groups, respectively, at the end of treatment visit (four weeks). A significant difference in the performance of both the drugs became apparent from second week. The difference in proportion between groups was comparable [4.7% (-0.8 - 10.3); p = 0.1707 and 5.2% (-1.8 - 12.3); p = 0.1954] in PP and ITT population, respectively (Table 2).

**Table 2:** Efficacy endpoints at week 4 per-protocol population and intent to treat population OTE, Overall treatment effect.

Endpoint	Acotiamide n (%)	Mosapride n (%)	Difference (95% CI)	Acotiamide vs Mosapride (p-value)
(A) Per-Protocol Population				
Primary Endpoint				
Responder rate by using OTE	98.00	93.27	4.7 (-0.8 - 10.3)	0.1707
Secondary Endpoints Elimination Rate				
Postprandial fullness	15.00	9.62	-5.4 (-14.4 - 3.6)	0.2883
Upper abdominal bloating	16.00	14.42	-1.6 (-11.4 - 8.3)	0.8460
Early satiation	11.00	13.46	2.5 (-6.5 - 11.4)	0.6718
(B) Intent to Treat Population				
Primary Endpoint				
Responder rate by using OTE	95.15	89.91	5.2 (-1.8 - 12.3)	0.1954
Secondary Endpoints Elimination Rate				
Postprandial fullness	14.56	9.17	-5.4 (-14.1, 3.3)	0.2875
Upper abdominal bloating	15.53	13.76	-1.8 (-11.3, 7.8)	0.8462
Early satiation	10.68	12.84	2.2 (-6.5, 10.8)	0.6744

Secondary efficacy endpoints

Overall Treatment Effect (OTE)

The responder rates in PP population in the Acotiamide and Mosapride groups were 6% versus 2.88% [(-2.5, 8.8); p = 0.3247)], 38% versus. 40.38% [(-15.8, 11.0); p = 0.7751)], 87% versus 78.85% [(-2.1, 18.4); p = 0.1398)] and 98% versus 93.27% [(-0.8, 10.3); p = 0.1707)] each week, respectively. Responder rates in ITT population in the Acotiamide and Mosapride groups were 5.83% versus 2.75% [(-2.4, 8.5); p = 0.3215)], 36.89% versus 39.45% [(-15.6, 10.5); p = 0.7776)], 84.47% versus 76.15% [(-2.3, 18.9), p = 0.1676)], and 95.15% versus 89.91% (95% CI is -1.8, 12.3; p = 0.1954) at each week, respectively.

Elimination Rate

The elimination rate (post-prandial fullness, upper abdominal bloating, and ES) at week 4 in the Acotiamide and Mosapride groups were 15% versus 9.62% [(-14.4, 3.6); p = 0.2883)], 16% versus 14.42% [(-11.4, 8.3); p = 0.8460)], 11% versus 13.46% [(-6.5, 11.4); p = 0.6718)] in PP population and 14.56% versus 9.17% [(-14.1, 3.3); p = 0.2875)], 15.53% versus 13.76% [(-11.3, 7.8); p = 0.8462)], and 10.68% versus 12.84% [(-6.5, 10.8); p = 0.6744)] in ITT population. The elimination rates were comparable between both the treatment groups (Table 2).

Improvement of Individual Symptom Scores

There was a notable improvement in both Acotiamide and Mosapride groups in individual symptom severity on a severity scale of 0 to 3 (none, mild, moderate, and severe) each week. There was no significant difference observed for the difference in improvement rates in individual symptom severity between the Acotiamide and Mosapride groups (Table 3).

**Table 3:** Individual symptom severity score (ISSS) - change from baseline to week 4 in ITT and PP population N, Total number of subjects; ITT, intent to treat; PP, per-protocol population.

Parameter	PP Population					ITT Population				
	Acotiamide [N = 100] Mean ± SD	Mosapride [N = 104] Mean ± SD	Acotiamide vs Mosapride			Acotiamide [N = 107] Mean ± SD	Mosapride [N = 111] Mean ± SD	Acotiamide vs Mosapride		
			Mean difference	95% CI	p-value			Mean difference	95% CI	p-value
Upper abdominal pain	1.8 ± 2.36	2.3 ± 2.78	-0.31	-1.8 - 1.1	0.1385	2.0 ± 2.63	2.6 ± 3.13	-0.41	-1.8 - 1.0	0.0954
Upper abdominal discomfort	0.4 ± 0.37	0.5 ± 0.40	-0.06	-0.3 - 0.2	0.1210	0.4 ± 0.43	0.5 ± 0.43	-0.07	-0.3 - 0.2	0.1549
Postprandial fullness	0.5 ± 0.37	0.5 ± 0.39	-0.08	-0.3 - 0.1	0.2718	0.5 ± 0.41	0.6 ± 0.48	-0.11	-0.3 - 0.1	0.1416
Upper abdominal bloating	0.4 ± 0.37	0.5 ± 0.45	0.03	-0.1 - 0.2	0.6627	0.5 ± 0.44	0.5 ± 0.52	0.01	-0.2 - 0.2	0.5608
Early satiation	0.5 ± 0.41	0.5 ± 0.45	-0.13	-0.3 - 0.0	0.4363	0.5 ± 0.47	0.6 ± 0.50	-0.13	-0.3 - 0.0	0.4234
Excessive belching	0.2 ± 0.36	0.2 ± 0.34	-0.08	-0.3 - 0.2	0.8176	0.3 ± 0.39	0.3 ± 0.39	-0.10	-0.3 - 0.1	0.5538
Nausea	0.1 ± 0.23	0.1 ± 0.21	-0.09	-0.3 - 0.1	0.2591	0.1 ± 0.26	0.2 ± 0.24	-0.10	-0.3 - 0.1	0.2003
Vomiting	0.1 ± 0.17	0.1 ± 0.16	-0.07	-0.2 - 0.0	0.3984	0.1 ± 0.22	0.1 ± 0.19	-0.08	-0.2 - 0.0	0.2723
Heartburn	0.1 ± 0.20	0.1 ± 0.21	-0.01	-0.2 - 0.2	0.9973	0.1 ± 0.23	0.1 ± 0.21	-0.01	-0.2 - 0.1	0.9488
Total individual symptoms score	2.2 ± 1.90	2.5 ± 1.96	-0.50	-1.5 - 0.6	0.2700	2.5 ± 2.28	2.8 ± 2.37	-0.59	-1.6 - 0.5	0.2229

Quality-of-life scores

The change in SF-NDI scale score in the Acotiamide and Mosapride groups was -13.9 versus -12.8 in the PP population and -13.7 versus -12.4 in the ITT population (Table 4). Based on the SF-NDI scores, the QoL had significantly improved (p < .0001) compared to baseline. The difference in SF-NDI scores [-1.15 (-2.4 - 0.1), p = 0.0693) in PP population and [-1.37 (-2.7 - 0.1), p= 0.0347)] in ITT population was comparable between Acotiamide and Mosapride.

**Table 4:** Summary of overall and subscale symptom scores on the Short Form-Nepean Dyspepsia Index (SF-NDI) questionnaire N, Total number of subjects; ITT, intent to treat; PP, per protocol; p-value < 0.0001.

	Change from baseline to Week 4 for SF-NDI (Score), mean ± SD			
Variable	PP Population (N = 204)		ITT Population (N = 218)	
	Acotiamide (N = 100)	Mosapride (N = 104)	Acotiamide (N = 107)	Mosapride (N = 111)
Overall symptom score	-13.9 ± 4.64	- 12.8 ± 4.71	-13.7 ± 4.67	-12.4 ± 4.94
Tension	-1.6 ± 0.69	-1.4 ± 0.63	-1.5 ± 0.71	-1.4 ± 0.66
Interference with daily activities	-3.2 ± 1.29	-3.0 ± 1.22	-3.1 ± 1.30	-2.9 ± 1.28
Eating/Drinking	-3.2 ± 1.27	-2.9 ± 1.36	-3.2 ± 1.28	-2.8 ± 1.40
Knowledge/Control	-3.0 ± 1.35	-2.8 ± 1.39	-3.0 ± 1.36	-2.7 ± 1.40
Work/Study	-2.9 ± 1.30	-2.7 ± 1.32	-2.9 ± 1.32	-2.6 ± 1.35

Safety

Overall, three adverse events (AEs) were reported in three patients receiving Acotiamide, and two adverse events were reported in two patients in the Mosapride group. All the five AEs were mild in severity, unlikely related to the study drug, wherein the subjects recovered without any sequelae (Table 5).

**Table 5:** Overall summary of treatment-emergent adverse events - safety population

System Organ Class Preferred Term	Acotiamide [N = 108] n (%)	Mosapride [N = 112] n (%)	Overall [N = 220] n (%)
Any treatment-emergent adverse event	3 (2.8)	2 (1.8)	5 (2.3)
Nervous system disorders			
Headache	1 (0.9)	1 (0.9)	2 (0.9)
Dizziness	0	1 (0.9)	1 (0.5)
Skin and subcutaneous tissue disorders			
Pruritus	2 (1.9)	0	2 (0.9)

## Discussion

FD is a common disorder that affects the upper digestive tract. It is a highly prevalent chronic gastrointestinal disorder that considerably lowers the QoL of affected patients and leads to frequent medical consultations [16]. The prevalence of FD among the communities may be higher than 20%. The symptoms associated with FD include epigastric pain, burning sensation, bloating, postprandial fullness, blanching, bloating, and nausea. Most affected people include those aged over 60 years as compared to young people and those who are potentially infected with Helicobacter pylori [17,18]. In general, FD patients are treated with proton pump inhibitors (omeprazole, esomeprazole, lansoprazole, dexlansoprazole, pantoprazole, and rabeprazole), neuromodulators (tricyclic antidepressants, serotonin reuptake inhibitors, serotonin noradrenaline reuptake inhibitors, and noradrenergic and specific serotonergic antidepressant), and prokinetic drugs (metoclopramide, cisapride, domperidone, ranitidine, Mosapride, metoclopramide, trimebutine, itopride, and Acotiamide) [19,20,21].

Currently, the recommended first-line treatments for FD are acid-suppressive or prokinetic drugs [22]. While prokinetics are recommended as initial therapy for treating PDS, there is still no consensus regarding the drug of choice [23,24]. Acotiamide is a prokinetic drug that has recently been approved in Japan for the treatment of FD. In phase II studies conducted in Japan and Europe, Acotiamide exerted gastroprokinetic activity, improved gastric emptying, and accommodation, thereby confirming its beneficial effects in alleviating FD symptoms that include postprandial fullness, upper abdominal bloating, and early satiation [21,23]. Improvement of FD symptoms in 93% of patients approximately after four weeks of administration of Acotiamide was reported by Behera et al. [25]. Narayanan et al. reported complete relief or significant improvement from postprandial fullness, upper abdominal bloating, and ES in 79.2%, 74.4%, and 77.1% patients, respectively (p < 0.001 for all versus no/slight improvement) when treated for >28 days or 14-28 days with Acotiamide [26]. Matsueda et al. reported a responder rate based on the OTE by 52.2% patients receiving Acotiamide 100 mg TID and 34.8% patients receiving placebo (p < 0.001). At the end of four weeks, the Acotiamide group (p = 0.004) showed improvement in all three meal-related FD symptoms. The elimination rate of all three meal-related symptoms (postprandial fullness, upper abdominal bloating, and early satiation) was 15.3% with Acotiamide and 9.0% with placebo (p = 0.004) [27].

Interestingly, most previous clinical studies have evaluated the performance, safety, and efficacy of Acotiamide in comparison to a placebo. There have been hardly any studies that compared Acotiamide with other similar drugs in the literature. A recent meta-analysis study had evaluated the efficacy of several drugs that are currently being used to treat FD. It was noted that among all the drugs tested during the randomized control trials, Acotiamide showed greater efficacy as compared to the placebo [28]. This study also is another proof of the fact that there are scanty reports of clinical trials carried out to assess the efficacy and safety of Acotiamide in comparison with an already existing drug instead of a placebo.

The long-term safety concerns associated with the use of Acotiamide were previously investigated. It was revealed that there was no clinical or laboratory result associated with noticeable adverse effects among people even after 50 weeks of administration [13]. The results of the current study showed that Acotiamide 100 mg TID had a consistent efficacy (OTE: 95.15%) concerning the improvement in individual FD symptoms (postprandial fullness: 14.56%; upper abdominal bloating: 15.53%; and early satiation: 10.68%). The study results also demonstrate significant improvement on all sub-domains of the disease-specific SF-NDI QoL assessment (overall symptom score: -13.7 ± 4.67), which is comparable to the comparator drug and the efficacy data of Acotiamide drug reported in the literature.

Being a functional disease, the endpoints in the study are subjective and are based on patient-reported outcomes on standardized response scales. However, the subjectivity of the response parameters could be a limiting factor in the study. Although the prevalence of the disease is widespread and often undertreated, this disease is not commonly and adequately diagnosed in the general population. Additionally, a longer duration of treatment is needed for long-term benefits.

## Conclusions

FD is one of the most common upper gastrointestinal disorders and is highly prevalent worldwide. FD symptom management remains challenging, thus comprising an unmet medical need. In the present study, Acotiamide 100 mg (hetero) TID showed a significant improvement in the QoL with consistent efficacy and safety that was comparable with Mosapride in Indian adult patients with FD. Acotiamide could be chosen as an alternative drug by physicians while treating patients with FD-PDS in India.
